# A Medical Worthy of the Early Eighteenth Century

**Published:** 1910-12-24

**Authors:** 


### A MEDICAL WORTHY OF THE EARLY EIGHTEENTH CENTURY.

The memoirs of Dr. Poumiès de la Siboutie have recently been published by his daughters. Dr. Léon Mac-Auliffe gives an interesting *résumé* of the worthy physician's "souvenirs" in La Clinique.* These were the result of a period of enforced rest, due to a fall by which he broke his leg. Born in Périgord in 1789, the young Poumiès came to Paris in 1810 to study medicine. Being scantily provided with the good things of this life, he made up for this shortcoming by the possession of good health and exuberant spirits. "A characteristic circumstance of this period," writes he, "was the great proportion of lame, deformed, and otherwise disfigured students. The same was also true of other classes of society. Conscription, voluntary engagement, and the military schools took away the strongest and best-looking young men, leaving in the family only those who were feeble, small, debilitated, or affected with some infirmity which rendered them unfit for service. We may say in passing that this circumstance has exercised in France a great influence on the population: the sons of these men have inherited these imperfections, and individual strength, the mean stature, and physical perfection have suffered a great diminution. Each year has necessitated the lessening of the standard of height insisted on for a would-be soldier." Poumiès learned his anatomy from Marjolin. At that time each professor had his own dissecting establishment, situated in a house the rooms of which were furnished with long tables destined for the reception of the bodies obtained from the hospitals at a cost of twenty francs per subject. Four students were allotted to each table, and took it in turns to dissect, while one of them read a *viva voce* description from a text-book. In 1812 Poumiès was named "interne" to the Paris Hospitals. For his services he received £20 per annum, and in addition board and lodging on his days of duty. He worked at the Salpêtrière under Pinel, "*le liberateur des alienés*," of whom he gives an appreciation that redounds to the credit of his chief. That same year he followed the obstetrical course of Capuron, held in an apartment which now forms part of the Musée de Cluny. He gives the following description of the practical side of such lectures: "When a woman is on the point of delivery she is taken to the amphitheatre, into which the students, previously warned, rush to secure places on the benches. The woman is made to lie down on the bed of pain in the middle of the room, so that everyone can see her; she is, however, treated with the greatest respect and kindness. The professor explains each stage of labour and the methods proper to the treatment of each. The birth having taken place, the woman is taken back to her home in a carriage, visited and helped until her recovery, receiving in addition the monetary reward previously agreed upon." The surgeon at the Salpêtrière was Lallemont, a weak-willed individual who was frightened of everything. His visits were always of long duration, and in urgent cases his "interne" knew how to bring these visits to an end quickly. For this he revived the method of Beaumarchais. "Are you unwell, sir?" he would say on the surgeon's arrival. "Why do you ask?" "You appear from your face to be tired." "You think so? Well, I will cut my visit short, although I do not feel ill." Poumiès followed Cuvier's course at the Jardin des Plantes. Of him he writes as follows: "His was the best organised head in France. He never lost a moment. His carriage was a travelling study furnished with a lamp for working at night. While dressing he always had one of his students present to report on some piece of work or to read to him. Politics and public affairs, to which he devoted himself later, quickly became familiar to him. He talked administration, jurisprudence, diplomacy as though he had done nothing else all his life. He was the soul of the Academy of Science, and few months passed without his publishing some important memoir. What a man, what a professor! He drew with rare perfection and great facility the animals about which he discoursed in his lectures; a piece of white chalk sufficed him for a faithful reproduction on the blackboard. . . . He died of apoplexy." At the Hôtel Dieu, Poumiès was "interne" to Dupuytren. He describes him as being little acquainted with the science of his time, but a very hard worker and of great practical skill. "That which he did not know he divined, he invented; however serious and unusual the circumstances, his genius never failed him." He revealed under circumstances related by Poumiès a

* "Dr. Poumiès de la Siboutie : Souvenirs d'un Médecin de Paris" (Plon, Paris, 1910).

loyalty and greatness of character such as are rarely met with. At the same period Asselin was physician to the Hôtel Dieu. He was a splendid and learned man. His optimism led him to contract the singular habit of saying upon all occasions " nous arrangerous tout cela." " We will arrange all that " was his reply one day to a lady who complained of the bad weather they were experiencing. It was a peculiar epoch which transformed the medical and law schools into artillery companies. On February 7, 1814, the medical students were summoned to the school to take their part in the defence of Paris. The disfavour into which Napoleon had fallen was then at its height. The humiliation of being beaten and the discomforts of a military occupation were as yet unknown. The youth of the city were wanting in their duty, and when the Comte de Lespinasse commenced to read his appeal to arms he was obliged to retire before a volley of cries, oaths, and challenges. In 1831 there was a menace of cholera, and at the end of March in the following year an outbreak occurred in Paris. Poumiès saw more than a hundred cases at the Hôtel Dieu, but most of them only once, for the disease lasted but a few hours. Mourning and consternation were general. The physicians were threatened, accused of being poisoners, and in some cases even assaulted. They carried out their duties notwithstanding, with but few exceptions. The authorities decided that gold medals should be awarded to a hundred physicians chosen from among those who had most distinguished themselves. Later it was decreed that the medals should be of silver to avoid any question of avarice. In the end, however, when the outbreak had been overcome, they were struck of copper! Poumiès was among the recipients. Dr. Mac-Auliffe gives us no further details of the life of this interesting character, but enough has been said to show the merits of the book to those interested in history of the times in which he lived.

